# Mothers’ Awareness Towards Infantile Colic in Saudi Arabia

**DOI:** 10.7759/cureus.50364

**Published:** 2023-12-11

**Authors:** Tareq H Alzahrani, Abeer M Anteet

**Affiliations:** 1 Pediatrics, King Saud Medical City (KSMC), Riyadh, SAU; 2 Pediatric Gastroenterology, King Khalid University Hospital, Riyadh, SAU

**Keywords:** sociodemographics, saudi arabia, infantile colic, awareness, mothers

## Abstract

Background

Infantile colic is defined as severe pain in the abdominal region of a baby primarily due to gastrointestinal implications and is believed to self-resolve with time. Recently established Rome IV criteria of diagnosis state that infantile colic should be diagnosed if there are symptoms like excessive crying, irritability and fussiness. Hence, this study aimed to assess the level of maternal awareness towards infantile colic in Saudi Arabia and to explore the relationship between the level of awareness and different socio-demographic factors like age, gender, nationality, etc.

Methodology

A cross-sectional study was carried out in Saudi Arabia from February to May 2021. An online self-administered questionnaire via Google Forms was used as the primary data collection tool. The generated link was randomly shared on electronic social media platforms including Facebook, WhatsApp, Telegram, and Twitter.

Results

A total of 425 participants were finally enrolled in the study. One-third of the participants (n=141, 33.2%) were aged more than 40 years and 399 were married (93.9%). Out of a total of 20 points, the mean score of maternal awareness was found to be 13.6±2.5. One-third of the participants (n=143, 33.6%) thought that rocking or carrying the baby would soothe the colic symptoms. Further, 175 participants (41.2%) used pain-relieving drugs and 7.8% sang lullabies. A total of 346 (81.4%) usually got frustrated/exhausted due to excessive crying sessions of the baby. Additionally, those who had received guidelines, educational programs or awareness sessions about the management of colic symptoms in babies had a significant awareness level (P-value = 0.032), while those who had not received education had poorer awareness.

Conclusion

Nearly one-third of the participants had good knowledge about infantile colic. More than one-third of the participants had previously received educational programs or awareness sessions about the management of colic symptoms in babies. More than half of the participants stated that postnatal maternal depression can occur as a result of infantile colic thereby psychological conflicts occur regarding the maternal role and inconsistent interaction styles with babies. Age, nationality, and marital status did not have a significant effect on the awareness level of the participants.

## Introduction

The word ‘colic’ generally refers to severe pain in the abdominal region primarily due to gastrointestinal implications such as increased gas in the intraluminal part or obstructions in the intestine. For instance, evidence-based recommendations for health and care in the UK provided by the National Institute for Health and Care Excellence (NICE) recommended that infantile colic might reflect a neurodevelopmental stage causing an exacerbation of normal infant crying due to abnormal gastrointestinal motility and gas production, reduced gut microbiome diversity, central nervous system deregulation, and psychosocial factors. Infantile colic refers to a condition in newly born babies and infants that is characterized by excessive crying without an obvious cause or ailment. This condition is considered benign in nature and is supposed to self-resolve after a given period of time. Mostly, infantile colic starts from the age of 2-3 weeks and resolves on its own at the age of 3-4 months [1].

Infantile colic is quite commonly prevalent in almost all parts of the world. It is estimated that almost 5%-40% of infants from around the globe are affected by this condition. Moreover, 10-20% of the medical care-seeking visits to hospitals or pediatricians in the early age of an infant are primarily presented with complaints of colic in babies. Most severe symptoms of colic are observed around the age of six weeks. Almost 60% of infantile colic cases self-resolve around the age of three months, while 90% of cases get resolved as soon as the baby reaches the age of four months [2].

The primary symptom of infantile colic is excessive crying without an obvious reason. In this condition, babies tend to cry with red face, move their legs in the upward direction and stretch their bellies. According to the classical standard diagnostic criteria, known as Wessel's Rule of 3s, infantile colic is diagnosed if the baby is fussy and cries for more than 3 hours per day for three or more days in a week, and this condition continues for three or more weeks starting from the third week of birth [3]. Recently established Rome IV criteria of diagnosis states that infantile colic should be diagnosed in a baby if symptoms like excessive crying, irritability and fussiness appear in a baby of less than five months of age, and the symptoms appear and disappear without any obvious cause or treatment. In addition to the stated diagnosis, there should not be another apparent medical ailment such as fever, weight loss or failure to thrive [4].

Despite the high incidence and global prevalence of infantile colic, the exact underlying mechanism that leads to the condition remains unidentified. There are many theories that point towards multiple etiologies, which include gastrointestinal mechanisms, psychological involvement and neurological implications. In addition, infantile colic is also considered to be caused by both physiological and psychological factors that might lead to exacerbation of normal infant behavior [5].

For the management of infantile colic, organic causes must be excluded before considering the confirmed diagnosis. However, many guidelines do not require organic causes to be investigated as the diagnosis can be obtained from the history. Some major organic causes for infant crying and irritability might include allergens in the maternal diet in the case of breastfed infants, intussusception, hair tourniquets around fingers or toes, foreign object in the eye, chest/nose congestion, injury or underlying medical disease, e.g., gastrointestinal reflux disease, lactose intolerance, hernia or any infection [6].

Like the causes, treatment options are not clearly defined due to mixed outcomes of the research conducted to analyze the efficacy of various interventions and therapies. The most effective treatment of infantile colic in exclusively breastfed babies could be the introduction of the probiotic strain Lactobacillus reuteri DSM 17938 [7]. Removing food items that might contain potential allergens such as fish, dairy items, tree nuts, etc. from the diet of nursing mothers has also proven to be possibly effective. However, it is not recommended to use L. reuteri in formula-fed babies, and hydrolyzed formulas are suggested instead. Moreover, removing noise and intense light sources from the environment might also help to reduce crying. In addition, regular massage by the mother and sucrose solution might also give temporary relief to the colic babies. In contrast, traditional approaches such as swaddling, giving herbal mixtures or using therapeutics like dicyclomine and cimetropium might provide temporary relief. Car riding, excessive carrying, rocking, crib vibrator and simethicone usage have been proven ineffective for infantile colic management [8].

Although infantile colic is itself a benign and self-limiting condition, it has been associated with maternal or caregiver depression and shaken baby syndrome. Parental reassurance is a critical step to avoid any unwanted outcomes that might lead to dangerous health conditions. Parents and caregivers are advised to lay down the baby back on the cot and walk away if they get overly irritated. It is not recommended to rock the baby when the mother or caregiver is frustrated because the baby might be harmed due to rigorous shaking or patting. Reassuring parents about the self-limiting nature of the condition, listening to their worries, appreciating their struggles, encouraging their efforts and educating them with possible coping strategies during crying sessions could be one of the most important management strategies and might help in better health outcomes in infants as well as in parents and caregivers [9].

Several studies have been conducted to elucidate the maternal awareness about infantile colic and coping strategies in different parts of the world. A randomized controlled trial carried out in Canada reported significant improvement in mothers who received educational intervention about infantile colic management and the dangers of shaken baby syndrome due to frustration from excessive baby crying [10]. In addition, a study from the Netherlands identified a link between mothers’ mental health and infants’ gastrointestinal health implying that an improved mother-infant bond can help in relieving gastric problems in babies [11]. Moreover, a study from Australia indicated common behavior of food avoidance in breastfeeding mothers to reduce the severity of infantile colic [12]. The perception of infantile colic among German and Polish pediatricians was analyzed in a study carried out in Poland. The outcome indicated that the majority of German pediatricians were well informed about infantile colic-associated health burden as well as the risk of maternal depression and shaken baby syndrome, while 50% of Polish pediatricians had average knowledge about the subject [13]. Subsequently, Nigerian mothers were assessed for their understanding of infantile colic in a cross-sectional study, which highlighted the need for parental education to avoid unnecessary medication in colic babies [14]. Furthermore, a dual support system encompassing colic management as well as social support and care was suggested by a qualitative exploratory descriptive study that was carried out in Brazil [15].

In Asia, some studies have indicated a significant maternal role in the management of infantile colic. For instance, the positive effects of social support and spousal assistance in the management and caretaking of colic babies have been described in a cross-sectional Iranian study [16]. Moreover, a study enrolling more than 200 Turkish mothers revealed that the majority (more than 60%) of mothers used behavioral coping mechanisms such as rocking, holding and messaging baby bellies and feet to manage infantile colic during 1-6 months of age [17]. Similarly, another study from Turkey revealed the effective nature of reflexology therapy to reduce the severity of colic symptoms in babies [18]. Additionally, the need for education in mothers about the management of colic babies was highlighted in an Indian study that indicated a lack of awareness in 78% of mothers about breastfeeding and newborn care [19].

Moreover, a study from Middle Eastern and North African countries including Kuwait, Lebanon, Saudi Arabia, Iraq and Morocco revealed that more educational and strategic interventions are required to establish management protocols for infantile colic in these countries [20]. Likewise, a strong need for health education regarding infantile colic management in mothers of infants was highlighted by a study carried out in Riyadh city of Saudi Arabia [21]. Moreover, a review-based study from Makkah City, Saudi Arabia, also emphasized the need for educational health awareness programs for the improvement in understanding the coping strategies to deal with crying babies and the management of colic symptoms in infants [22].

Hence, this study was carried out with the aim to study the level of mothers’ awareness towards infantile colic in Saudi Arabia and explore the relationship between the level of awareness and different socio-demographic factors.

## Materials and methods

Study design and location

A cross-sectional study was carried out in different regions of Saudi Arabia from February to May 2021.

Inclusion and exclusion criteria

Any mother, who was a resident of Saudi Arabia, agreed to participate in the study, had any nationality, could read, and had a social media account was included in the study. Whereas, non-Saudi Arabian residents, who had no social media account, and those who refused to participate in the study were excluded. Further, infants with complicated medical surgical history were also excluded.

Sample size

To ensure the reliability of the study outcomes, the sample size necessary for investigating maternal awareness was determined using the EPI info program, based on a 95% confidence interval, 5% margin of error, and the total population of mothers in Saudi Arabia obtained through the data from the Saudi General Authority for Statistics. The estimated sample size was found to be 384 and was adjusted to 422 to compensate for 10% non-response rate.

Data collection tool

An online self-administered questionnaire via Google Forms was used as the primary data collection tool. The generated link was randomly shared on electronic social media platforms including Facebook, WhatsApp, Telegram, and Twitter. The aim of the study was clearly explained in the interface.

A validated questionnaire was used based on previous studies [10, 14, 17, 19, 21]. The questionnaire contained socio-demographic characteristics of the participants like age group, nationality, and residence. The questionnaire also included questions about mothers’ awareness towards infantile colic.

A common grading method was applied to quantifying each variable, a score of 2 points for accurate answers, 0 for incorrect answers and 1 point for neutral ones. The scores were summed up post data collection and participants scoring 75% or more questions (15 points out of 20) were considered to exhibit substantial awareness of infantile colic.

Pilot study

The questionnaire was pretested in a pilot study over a sample of 20 participants whose results were not included in the study. Some modifications were done accordingly first in English language and then it was translated to Arabic to ensure clarity and easy understanding of the questions.

Sampling technique

A convenient non-probability sampling technique was employed for data collection.

Data analysis

Data were coded, entered, and analyzed using the Statistical Package for Social Sciences (SPSS) version 23 (IBM Corp., Armonk, NY, USA). Qualitative data were expressed in the form of numbers and percentages (No. & %). The chi-square (χ2) test was used to examine qualitative data between the two groups.

Ethical considerations

Respective approval of the study was obtained from the Research Ethics Committee in King Saud University Medical City via reference number 21/0082/IRB, dated 16/01/2021. All participants were volunteers and their data were kept confidential and were used only for research purposes.

## Results

A total of 425 participants were finally enrolled in the study. One-third of the participants (n=141, 33.2%) were aged more than 40 years, 415 (97.6%) were from Saudi Arabia and 399 were married (93.9%). More than half of them (n=264, 62.1%) had bachelor’s degrees. A total of 178 (41.9%) were employed (Table 1).

**Table 1:** Socio-demographic characteristics of the participants (n=425)

Variable	Category	Frequency	Percentage
Age	< 20 years	7	1.6
	21-25 years	24	5.6
	26-30 years	48	11.3
	31-35 years	68	16
	36-40 years	137	32.2
	> 40 years	141	33.2
Nationality	Saudi	415	97.6
	Non-Saudi	10	2.4
Marital status	Single	8	1.9
	Married	399	93.9
	Divorced	11	2.6
	Widowed	7	1.6
Number of children	1	40	9.4
	2	70	16.5
	3	66	15.5
	4	99	23.3
	5	75	17.6
	> 5	75	17.6
Age of the eldest child	< 1 year	14	3.3
	1-2 years	13	3.1
	2-3 years	11	2.6
	3-4 years	11	2.6
	4-5 years	16	3.8
	> 5 years	360	84.7
Age of the youngest child	< 1 year	66	15.5
	1-2 years	51	12
	2-3 years	44	10.4
	3-4 years	30	7.1
	4-5 years	57	13.4
	> 5 years	177	41.6
Education	Primary	17	4
	Intermediate	24	5.6
	Secondary	82	19.3
	Bachelors	264	62.1
	Other	38	8.9
Occupational status	Employed	178	41.9
	Unemployed	200	47.1
	Retired	21	4.9
	Private business	11	2.6
	Other	15	3.5
Average monthly income (SAR)	Less than 3,000	38	8.9
	3,000 – 4,999	60	14.1
	5,000 – 9,999	131	30.8
	10,000 – 15,000	116	27.3
	More than 15,000	80	18.8

**Figure 1:**
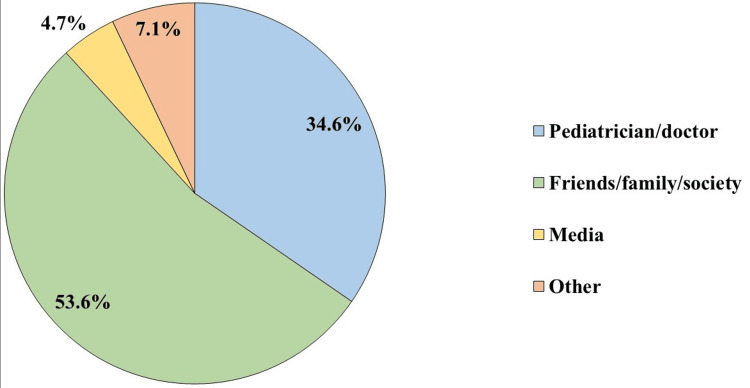
Source of participants' information about colic in infants (n=425)

Out of a total of 20 points, the mean score of mother’s awareness was found to be 13.6 ± 2.5, with a minimum value of 6 and a maximum value of 19. More than half (n=271, 63.8%) of participants had poor knowledge and 154 (36.2%) had good knowledge. The majority of the participants (n=408, 96.2%) had heard about the condition of infantile colic. The source of information was diverse, with about half (n=228, 53.6%) of the participants relied upon friends/family and society (Figure 1).

**Figure 2:**
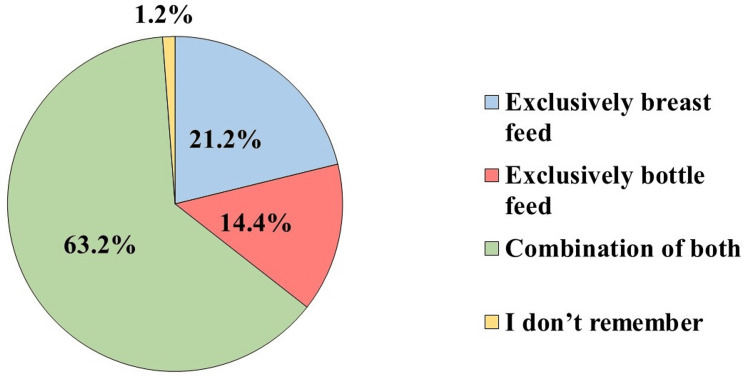
Feeding method of baby/babies for initial six months of age (n=425)

A total of 168 (39.5%) participants had received educational programs or awareness sessions about the management of colic symptoms in babies after baby birth. And 269 (63.3%) preferred combination feed for the initial six months of age (Figure 2).

**Figure 3:**
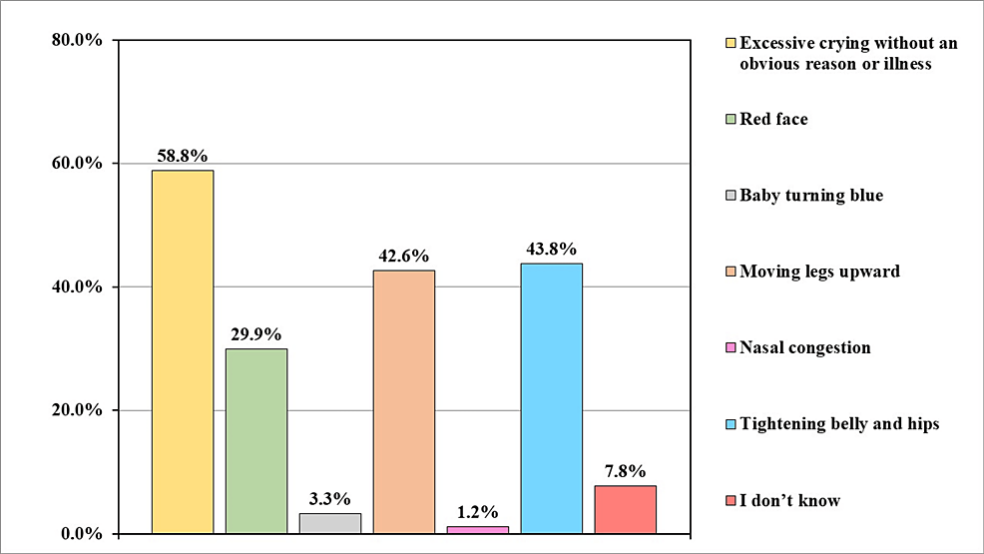
What are the primary symptoms of infantile colic? (n=425)

Regarding the primary symptoms of infantile colic, more than half (n=250, 58.8%) of the participants recognized excessive crying without an obvious cause as a potential symptom, 186 (43.8%) said tightening of belly and hips, and only 14 (3.3%) said baby turning blue (Figure 3).

**Figure 4:**
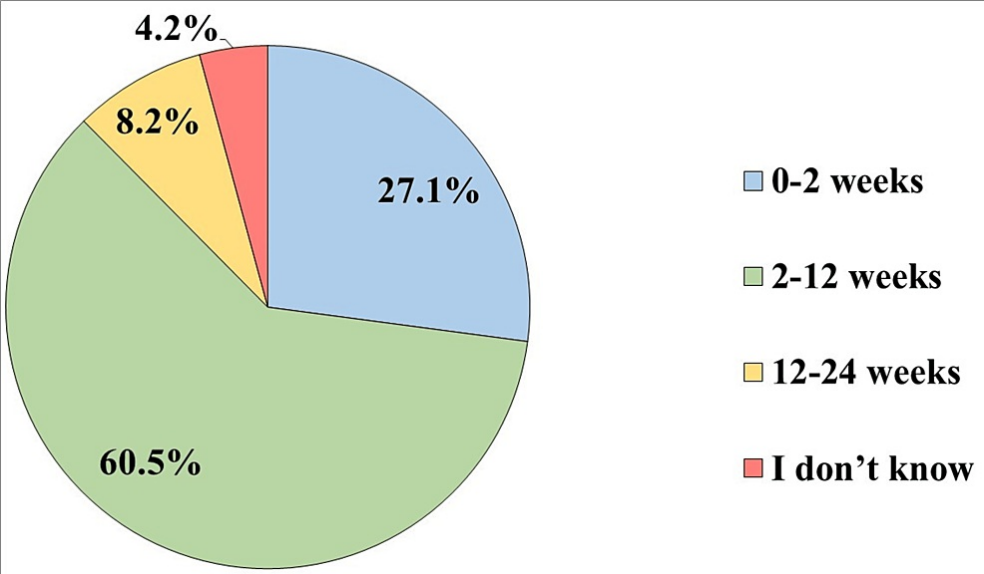
Which age group of infants is usually affected with infantile colic? (n=425)

Regarding the age of infantile colic, 257 (60.5%) reported the age group of 2 to 12 weeks as the most susceptible to infantile colic (Figure 4).

**Figure 5:**
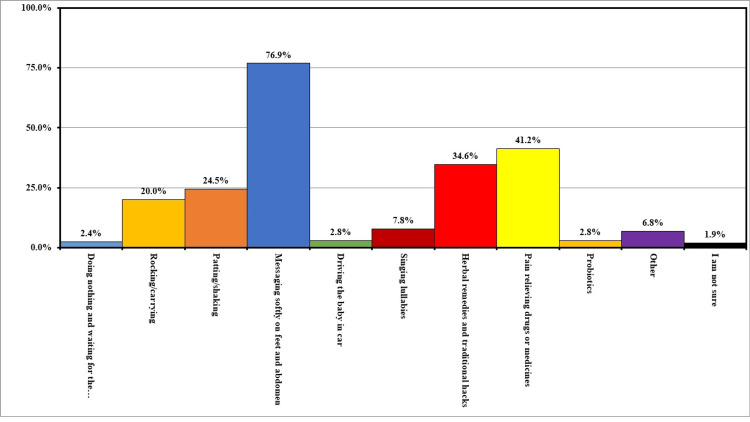
What is your most likely chosen method to calm down a colic baby?

More than half of the participants (63.8%) were unaware that infantile colic pain is dangerous for babies. A total of 267 (62.8%) participants perceived colic to self-resolve after 3-4 months of age, and 193 (45.4%) participants were certain that herbal remedies were safe to resolve colic problems. Furthermore, one-third (n=143, 33.6%) thought that rocking or carrying the baby could soothe the colic symptoms. A total of 389 (91.5%) thought that warmth/closeness with the mother and/or a massage by the mother can help in relieving the symptoms of colic. More than two-thirds (76.9%) of participants did soft massaging of abdomen and feet. While 175 (41.2%) participants used pain-relieving drugs (Figure 5).

**Figure 6:**
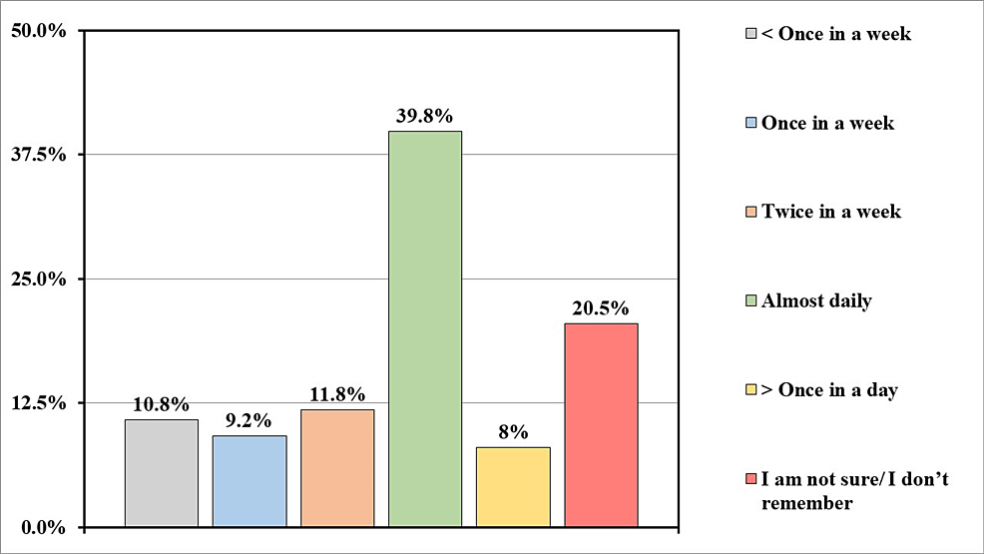
How often do you feel you need a break when taking care of a colic infant?

A total of 346 (81.4%) participants got frustrated/exhausted due to excessive crying sessions of the baby. And 219 (51.5%) participants were aware of postnatal maternal depression. While 219 (51.5%) participants agreed that excessive crying in colic babies can cause maternal depression. More than half of the participants (n=285, 67%) had received support for taking care of the baby from family/spouse/society/healthcare workers. And 169 (39.8%) participants expressed that a break from baby care on a daily basis was needed (Figure 6).

A total of 330 (77.6%) participants did not know about shaken baby syndrome. While 219 (51.5%) participants agreed that a frustrated/depressed/exhausted mother could unintentionally hurt a baby affected with infantile colic (Table 2 and Table 3).

**Table 2:** Mothers’ awareness towards infantile colic

Question	Yes, N (%)	No, N (%)	I don’t remember, N (%)
Have you ever heard about the condition Infantile colic?	409 (96.2)	9 (2.1)	7 (1.6)
Did you receive any guidelines, educational program or awareness session about the management of colic symptoms in babies after baby birth?	168 (39.5)	247 (58.1)	10 (2.4)

**Table 3:** Mothers’ awareness towards infantile colic

	Yes, N (%)	No, N (%)	I am not sure, N (%)
Do you think colic in babies is a dangerous condition?	37 (8.7)	271 (63.8)	117 (27.5)
Do you think colic in infants can resolve on its own after 3-4 months of age?	267 (62.8)	47 (11.1)	111 (26.1)
Do you think herbal remedies are safe for infants to resolve colic problems?	193 (45.4)	112 (26.4)	120 (28.2)
Do you think rocking or carrying the baby can soothe the colic symptoms?	143 (33.6)	206 (48.5)	76 (17.9)
Do you think warmth/closeness with the mother and/or a massage by the mother can help in relieving the symptoms of colic?	389 (91.5)	8 (1.9)	28 (6.6)
Do you usually get frustrated/exhausted due to excessive crying sessions of your baby?	346 (81.4)	64 (15.1)	15 (3.5)
Do you know about postnatal maternal depression?	306 (72)	95 (22.4)	24 (5.6)
Do you think excessive crying in colic babies can cause maternal depression?	219 (51.5)	95 (22.4)	111 (26.1)
Did you receive any help or support for taking care of the baby from family/spouse/society/healthcare workers?	286 (67.3)	123 (28.9)	16 (3.8)
Do you know about shaken baby syndrome?	57 (13.4)	330 (77.6)	38 (8.9)
Do you think a frustrated/depressed/exhausted mother can unintentionally hurt a baby affected with infantile colic?	219 (51.5)	98 (23.1)	108 (25.4)

Age was insignificant in affecting the awareness level of mothers (P-value = 0.574). Also, there was no significant association between nationality or marital status and awareness level (P-value = 0.114 and 0.759, respectively). The result also demonstrated that there was no significant association between marital status and the number of children with participant’s level of awareness (P-value = 0.759 and 0.335, respectively). Education, occupational status and average monthly income did not significantly affect the awareness of mothers (P-value = 0.688, 0.844, 0.823, respectively), but receiving guidelines, educational programs or awareness sessions about the management of colic symptoms in babies had a significant effect on the awareness level (P-value = 0.032) since those who did not receive education had poor awareness than others (Table 4).

**Table 4:** Factors associated with mothers’ awareness towards infantile colic

Variable	Category	Level of Awareness		P-value
		Poor, N (%)	Good, N (%)	
Age	< 20 years	4 (57.1)	3 (42.9)	0.574
	21-25 years	17 (70.8)	7 (29.2)	
	26-30 years	29 (60.4)	19 (39.6)	
	31-35 years	46 (67.6)	22 (32.4)	
	36-40 years	80 (58.4)	57 (41.6)	
	> 40 years	95 (67.4)	46 (32.6)	
Nationality	Saudi	267 (64.3)	148 (35.7)	0.114
	Non-Saudi	4 (40)	6 (60)	
Marital status	Single	4 (50)	4 (50)	0.759
	Married	255 (63.9)	144 (36.1)	
	Divorced	8 (72.7)	3 (36.1)	
	Widowed	4 (57.1)	3 (42.9)	
Number of children	1	30 (75)	10 (25)	0.335
	2	40 (57.1)	30 (42.9)	
	3	37 (56.1)	29 (43.9)	
	4	65 (65.7)	34 (34.3)	
	5	50 (66.7)	25 (33.3)	
	> 5	49 (65.3)	26 (34.7)	
Education	Primary	11 (64.7)	6 (35.3)	0.668
	Intermediate	17 (70.8)	7 (29.2)	
	Secondary	48 (58.5)	34 (41.5)	
	Bachelors	168 (63.6)	96 (36.4)	
	Other	27 (71.1)	11 (28.9)	
Occupational status	Employed	114 (64)	64 (36)	0.844
	Unemployed	124 (62)	76 (38)	
	Retired	15 (71.4)	6 (28.6)	
	Private business	7 (63.6)	4 (36.4)	
	Other	11 (73.3)	4 (26.7)	
Average monthly income (SAR)	Less than 3,000	26 (68.4)	12 (31.6)	0.832
	3,000 – 4,999	41 (68.3)	19 (31.7)	
	5,000 – 9,999	82 (62.6)	49 (37.4)	
	10,000 – 15,000	74 (63.8)	42 (36.2)	
	More than 15,000	48 (60)	32 (40)	
Received guidelines, educational program or awareness session about the management of colic symptoms in babies	Yes	95 (56.5)	73 (43.5)	0.032
	No	168 (68)	79 (32)	
	I am not sure	8 (80)	2 (20)	

## Discussion

Raising the mother’s knowledge and awareness about infantile colic would result in proper management of this condition as it affects healthy and well-fed infants below three months of age. Further, higher levels of knowledge would also help in the differentiation between infantile colic, which is simply a benign condition, and other serious conditions such as intestinal obstruction [23].

The aim of this study was to assess the level of mothers’ awareness towards infantile colic in Saudi Arabia and also to explore the relationship between the level of awareness and different socio-demographic factors.

It was found that one-third of the participants had good knowledge and awareness about infantile colic; this level of knowledge reported in the current study was considered to be higher when compared to another study conducted in Saudi Arabia by Al-Shehri et al., in which about 80% of participants had poor knowledge about infantile colic [21].

The vast majority of the participants had previously heard about the condition of infantile colic. The source of information and knowledge was from friends/family and society in more than half of the participants and this was found to be contradictory to the study which was conducted in Turkey in which the most reported source of information was the physician [24].

More than one-third of them had received educational programs or awareness sessions about the management of colic symptoms in babies after baby birth. Regarding infant feeding, nearly two-thirds of the participants were doing combined feeding of breast and bottle feeding for the initial six months of age. Concerning the primary symptoms of infantile colic, presentation with excessive crying without an obvious cause was mentioned by more than half of the participants, about less than half of them also mentioned tightening belly and hips, and moving legs upward; similar findings were reported in the study conducted in Belgium [6].

Further, the current study showed that two-thirds of the participants thought that colic in infants can resolve on its own after 3-4 months of age and this was supported by the findings mentioned in another study which was conducted in Sweden [8].

Slightly less than half of them mentioned that herbal remedies are safe for infants to resolve colic problems; another study conducted in Italy demonstrated the relationship between infantile colic and herbals and suggested low-quality evidence that herbals can resolve colic problems [25].

More than half of the participants stated that postnatal maternal depression can occur as a result of infantile colic, thereby psychological conflicts occur regarding the maternal role and inconsistent interaction styles with babies. These findings were found to be consistent with the findings reported in a parallel study which was conducted in Poland [13].

Two-thirds of the participants received support for taking care of a baby from family, spouse, society and healthcare workers. More than one-third of them felt that a break from childcare was needed almost daily. Half of them agreed that frustrated, depressed and exhausted mothers can unintentionally hurt a baby affected with infantile colic. More than two-thirds of them do not know about shaken baby syndrome. This percentage was higher when compared to another study conducted by Marcinkowska et al., in which less than half of the participants did not know about shaken baby syndrome [26].

Age, nationality, and marital status do not significantly affect the level of awareness of participants about infantile colic. Education, occupational status and average monthly income do not significantly affect the awareness of mothers (P-value = 0.688, 0.844, 0.823, respectively), and all this was found to be contradictory to another study conducted in Najran in Saudi Arabia, in which all previously mentioned socio-demographic factors were positively correlated to knowledge and awareness about infantile colic [27].

The study's cross-sectional design prevents establishing direct cause-and-effect relationships between socio-demographic factors and women’s awareness of infantile colic. Relying only on self-reported data might introduce biases and inaccuracies, potentially skewing the validity of the findings. Additionally, the focus on specific regions in Saudi Arabia might constrain the applicability of the results nationwide due to potential variations in cultural, socio-demographic factors, and healthcare access. To address these limitations, it would be beneficial to consider longitudinal studies to better grasp causal associations. Employing diverse data collection methods beyond self-reports could enhance accuracy. Furthermore, expanding the study's geographic scope across Saudi Arabia would provide a more comprehensive understanding of infantile colic awareness among women. Moreover, the use of an online questionnaire was a practical choice, though it limits the study to mothers with internet access and might introduce selection bias.

## Conclusions

Only one-third of the participants had good knowledge and awareness about infantile colic, and the majority of them had previously received educational programs or awareness sessions about the management of colic symptoms in babies. More than half of the participants stated that postnatal maternal depression can occur as a result of infantile colic, thereby psychological conflicts occur regarding the maternal role and inconsistent interaction styles with babies. Therefore, more health education is needed in order to increase the public knowledge and awareness level about infantile colic. This can be accomplished through health education campaigns and by supporting the role of media in the distribution of knowledge and awareness about infantile colic.
